# Ultrasmall Functionalized UiO-66 Nanoparticle/Polymer Pebax 1657 Thin-Film Nanocomposite Membranes for Optimal CO_2_ Separation

**DOI:** 10.1021/acsami.3c16093

**Published:** 2024-01-12

**Authors:** Lidia Martínez-Izquierdo, Cristina García-Comas, Shan Dai, Marta Navarro, Antoine Tissot, Christian Serre, Carlos Téllez, Joaquín Coronas

**Affiliations:** † Instituto de Nanociencia y Materiales de Aragón (INMA), Universidad de Zaragoza-CSIC, Zaragoza 50018, Spain; ‡ Chemical and Environmental Engineering Department, Universidad de Zaragoza, Zaragoza 50018, Spain; § Laboratorio de Microscopías Avanzadas, Universidad de Zaragoza, Zaragoza 50018, Spain; ∥ Institut des Matériaux Poreux de Paris, Ecole Normale Supérieure, ESPCI Paris, CNRS, PSL University, Paris 75005, France

**Keywords:** metal−organic framework (MOF), ultrasmall MOF, UiO-66, thin-film nanocomposite (TFN) membrane, gas separation

## Abstract

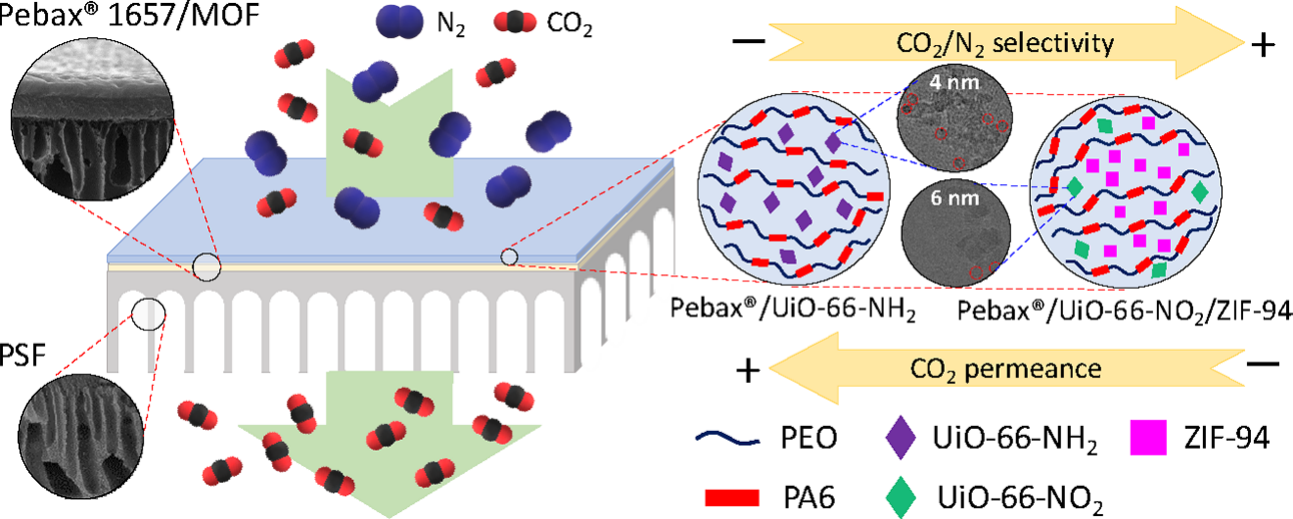

Ultrasmall 4 to 6 nm nanoparticles of the metal–organic framework (MOF) UiO-66 (University of Oslo-66) were successfully prepared and embedded into the polymer Pebax 1657 to fabricate thin-film nanocomposite (TFN) membranes for CO_2_/N_2_ and CO_2_/CH_4_ separations. Furthermore, it has been demonstrated that ligand functionalization with amino (−NH_2_) and nitro (−NO_2_) groups significantly enhances the gas separation performance of the membranes. For CO_2_/N_2_ separation, 7.5 wt % UiO-66-NH_2_ nanoparticles provided a 53% improvement in CO_2_ permeance over the pristine membrane (from 181 to 277 GPU). Regarding the CO_2_/N_2_ selectivity, the membranes prepared with 5 wt % UiO-66-NO_2_ nanoparticles provided an increment of 17% over the membrane without the MOF (from 43.5 to 51.0). However, the CO_2_ permeance of this membrane dropped to 155 GPU. The addition of 10 wt % ZIF-94 particles with an average particle size of ∼45 nm into the 5 wt % UiO-66-NO_2_ membrane allowed to increase the CO_2_ permeance to 192 GPU while maintaining the CO_2_/N_2_ selectivity at ca. 51 due to the synergistic interaction between the MOFs and the polymer matrix provided by the hydrophilic nature of ZIF-94. In the case of CO_2_/CH_4_ separation, the 7.5 wt % UiO-66-NH_2_ membrane exhibited the best performance with an increase of the CO_2_ permeance from 201 to 245 GPU.

## 1 Introduction

To achieve the Paris Agreement (reinforced in subsequent COPs) target of a decrease of 1.5 °C in the global average temperature increase, harmful greenhouse gas emissions must be considerably reduced in the coming decades. In order to accomplish this target, energy-efficient and low-carbon footprint technologies, as well as CO_2_ capture and storage approaches, must be developed. Membrane-based processes have emerged as attractive candidates for energy-efficient gas separations.^1^ However, dense membranes are not competitive for large-scale applications because of their very low CO_2_ permeance.^2^ Recently, the development of composite membranes with a very thin selective layer has attracted much attention due to their potential to achieve efficient separations, which exceed the permeance selectivity-inherent upper bound for a given separation, visualized for dense membranes as the so-called Robeson trade-off relationship between permeability and selectivity for a certain binary gas mixture.^3^ The formation of defect-free thin-film composite (TFC) membranes with a selective layer thickness lower than 1 μm may exhibit high CO_2_ permeances while maintaining or even increasing the selectivity. Such improvements over current dense films would make TFC membranes economically viable for implementation in the processing of power station flue gases.^4^

However, not only the membrane microstructure is of importance, but the polymer matrices used for CO_2_ separation also play a critical role.^5^ Block copolymers made of glassy and rubbery segments in different ratios are promising materials showing good performance in gas separation.^6,7^ Among them, poly(ether-*block*-amide) copolymers known under the trademark Pebax are being widely studied.^8−10^ These copolymers, comprising aliphatic polyamides and polyether segments, provide a high gas permeability and mechanical stability together with a high level of selectivity to CO_2_.^8^ Such is possible due to the introduction of polar groups with affinity to CO_2_ that improve CO_2_/nonpolar gas selectivity.^11^ Furthermore, the polymers can be enhanced with fillers to fabricate the so-called mixed matrix membranes (MMMs). Metal–organic frameworks (MOFs) with molecular sieving properties are regarded as promising filler crystalline porous materials to improve the separation performance of Pebax-based membranes.^12^ In addition to the nanostructured and adsorption properties of inorganic additives (e.g., amorphous porous silicates and zeolites), MOFs offer enhanced matrix compatibility and tunability due to the presence of organic linkers.^13^ In addition, the functionalization of MOFs with amino (−NH_2_), alkoxy (RO−), or nitro (−NO_2_) functional polar groups may improve their compatibility and adsorption properties for gases such as CO_2_.^14−16^ Anjum et al.^17^ reported an MMM made of polyimide Matrimid and UiO-66 (a Zr-MOF based on a benzenedicarboxylate ligand^18^) as a filler. They found that the preparation of the MOF with amine-functionalized linkers enhanced its intrinsic separation performance and improved the MOF–polymer compatibility. By contrast to the unfilled Matrimid membrane, they achieved a 50% more selective and 540% more permeable membrane when UiO-66-NH_2_ particles were used at a 30 wt % loading for CO_2_/CH_4_ separations. Qian et al.^19^ combined a high-molecular-weight polyimide of identical chemical structure to that of amine-functionalized UiO-66 nanoparticles to improve their interfacial compatibility. This strategy enabled them to create a defect-free MMM that is 48% more permeable and 18% more selective than the bare membrane in CO_2_/N_2_ separations.

In TFC membranes, as in MMMs, polymer enhancement can be done with the introduction of fillers to give what have been called thin-film nanocomposite (TFN) membranes. TFN membranes have been widely applied in nanofiltration and reverse osmosis processes,^20−22^ but their application to gas separation is scarce.^23,24^ MOF nanoparticles are essential to produce processable, high-performance TFN membranes where, due to the increase of the filler–polymer interfacial area, the mechanical and separation properties are enhanced with minor filler loadings (which, in turn, results in lower membrane cost). In this sense, MOF nanoparticles of UiO-66 have been widely used in TFN membranes for separations in the liquid phase,^25−27^ and only a few attempts have been made in the gas phase.^28−30^ In the case of gases, the development of TFN membranes requires the smallest possible fillers given the nanometric thickness of the selective layers and to avoid defects that are critical in gas separation. In this sense, particle agglomerations must be avoided, and the filler–polymer interaction must be improved. Recently, UiO-66-based MOFs have been synthesized as ultrasmall nanoparticles, e.g., 8–15 nm in the case of UiO-66-NH_2_^31^ and 4–6 nm in the case of UiO-66, UiO-66-NH_2_, and UiO-66-NO_2_.^32^ This last work by the authors (deposited in ChemRxiv) represents a new opportunity of membrane improvement, particularly in the form of TFN membranes where, as far as we are concerned, research on such ultrasmall nanoparticles has not yet been realized. Likewise, representing an efficient strategy to avoid filler agglomeration, combining two fillers of different nature in the same membrane has only been tested in MMMs and in TFN membranes for liquid separation^33−35^ but never in TFN for gas separation and even less involving ultrasmall MOFs.

In this work, we incorporate for the first time ultrasmall nanoparticles of amino (−NH_2_)- and nitro (−NO_2_)-functionalized UiO-66, exhibiting a record-low particle size ranging from 4 to 6 nm, into the Pebax 1657 polymer for the fabrication of TFN membranes for the separation of CO_2_/N_2_ and CO_2_/CH_4_ mixtures. Moreover, the possibility of combining the separation properties of these nanosized MOFs with those of nanoparticles of ZIF-94 has been explored. Like Pebax 1657, ZIF-94 is hydrophilic, and it is expected that the interaction between the polymer matrix and the MOFs will improve due to the enhanced interaction between Pebax and ZIF-94, which may also act as a binding agent for UiO-66 nanoparticles. Therefore, the nanoparticles of the two MOFs with different chemistry and structure will synergistically blend with the polymer, avoiding their agglomeration and increasing the MOF–polymer interfacial area. To our knowledge, the simultaneous formulation of ultrasmall functionalized UiO-66 and ZIF-94 nanoparticles in the same TFN membrane has never been explored in gas separation.

Other important aspects that may affect the gas separation performance of membranes are the content of water in the gas stream,^10^ the long-term stability,^36^ and the operation conditions (pressure and temperature).^6,37^ Nevertheless, this work only aims at studying the influence of ultrasmall nanoparticles of MOFs on the gas separation performance of TFN membranes since we consider that this important novelty needs a concentrated focus. Future work should be performed to elucidate the influence of these parameters on the gas separation performance of the optimum membranes fabricated in this work.

## 2 Experimental Section

### 2.1 Materials

Pellets of polysulfone (Udel P-3500 LCD) were purchased from Solvay Advanced Polymers. Poly[1-(trimethylsilyl)prop-1-yne] (PTMSP) was purchased from Fluorochem, United Kingdom. Polyether-*block*-amide, Pebax MH 1657 (comprising 60 wt % poly(ethylene oxide) (PEO) and 40 wt % aliphatic polyamide (PA6)) in the form of pellets was kindly provided by Arkema, France. 2-Aminoterephthalic acid (BDC-NH_2_) and anhydrous zirconium tetrachloride (ZrCl_4_) were purchased from Acros Chemicals. Zirconyl chloride octahydrate (ZrOCl_2_·8H_2_O) was purchased from Alfa Aesar. Zinc nitrate hexahydrate (Zn(NO_3_)_2_·6H_2_O) and 2-methylimidazole (2-mIm) were purchased from Sigma-Aldrich. 4-Methyl-5-imidazole-carboxyaldehyde was purchased from Acros Chemicals. The solvents methanol (MeOH), 1-butanol (1-ButOH), *N*-methyl-2-pyrrolidone (NMP), and absolute ethanol (EtOH) were purchased from Análisis Vinicos, Panreac, and Gilca, Spain. All gases used for the separation tests were of research grade (greater than 99.995% purity) and supplied by Abelló Linde S.A., Spain. All gases, polymers, reactants, and solvents were used as received.

### 2.2 Methods

#### 2.2.1 UiO-66 Synthesis and Functionalization with Nitro (−NO_2_) and Amino (−NH_2_) Groups^32^

##### 2.2.1.1 Synthesis of Zr_6_ Oxoclusters

ZrCl_4_ (2 g, 8.4 mmol) was added into a mixture of 3 mL of glacial acetic acid and 5 mL of isopropanol under stirring at 500 rpm while being heated at 120 °C for 60 min. The product was collected either through suction filtration or centrifugation at 10,000 rpm. The collected white solid was subsequently washed with acetone twice and dried under vacuum at room temperature (RT).

##### 2.2.1.2 Synthesis of Ultrasmall UiO-66

Zr_6_ oxoclusters (0.3 g) were dispersed in acetic acid (2 mL) under stirring at 600 rpm. H_2_O (5 mL) was subsequently added, and the reaction mixture was stirred until it became completely colorless. Ethanol (320 mL) was introduced into the solution followed by the immediate addition of benzene-1,4-dicarboxylic acid (200 mg, 1.2 mmol), and the reaction was stirred for 2 h at RT. The resulting solution was evaporated by rotary evaporation at RT until an approximately 50 mL volume was left. The colloidal suspension was centrifuged at 14,500 rpm for 45 min and then washed twice with the mixture of 30 mL of acetone and 30 mL of ethanol (14,500 rpm, 1.5 h). The collected solid was dried under vacuum for 3 h for characterization and application.

##### 2.2.1.3 Synthesis of Ultrasmall UiO-66-NH_2_

Zr_6_ oxoclusters (0.3 g) were dispersed in acetic acid (2 mL) under stirring at 600 rpm. H_2_O (5 mL) was subsequently added, and the reaction mixture was stirred until it became completely colorless. Ethanol (320 mL) was introduced into the solution followed by the immediate addition of 2-aminobenzene-1,4-dicarboxylic acid (220 mg, 1.2 mmol), and the reaction was stirred for 2 h at RT. The resulting solution was evaporated by rotary evaporation at RT until an approximately 50 mL volume was left. The product was recovered following the same procedure as for UiO-66.

##### 2.2.1.4 Synthesis of Ultrasmall UiO-66-NO_2_

Zr_6_ oxoclusters (0.3 g) were dispersed in acetic acid (2 mL) under stirring at 600 rpm. H_2_O (5 mL) was subsequently added, and the reaction mixture was stirred until it became completely colorless. Ethanol (80 mL) was introduced into the solution followed by the immediate addition of 2-nitrobenzene-1,4-dicarboxylic acid (250 mg, 1.2 mmol), and the reaction was stirred for 2 h at RT. The product was recovered following the same procedure as for UiO-66.

##### 2.2.1.5 Synthesis of 150 nm UiO-66-NH_2_

The synthesis protocol followed the reported paper^38^: 2 mmol (677 mg) of ZrOCl_2_·8H_2_O was weighted in a glass vial, and 7 mL of formic acid and 16 mL of distilled water were stepwise introduced in the reactor followed by 1 min of stirring at 600 rpm. Two mmol (352 mg) of 2-aminoterephthalic acid (BDC-NH_2_) and 20 mL of ethanol were subsequently added to the solution. The solution became a very cloudy solution after 12 h, indicating the formation of UiO-66-NH_2_. The product was recovered following the same procedure as for UiO-66.

#### 2.2.2 ZIF-94 Synthesis

ZIF-94 nanoparticles were synthesized via a solvent-assisted ligand exchange (SALE) reaction according to a previously reported method by Marti et al.^36,39^ Briefly, 0.323 g of 4-methyl-5-carboxyaldehyde (2.94 mmol) was first dissolved in 20 mL of 1-ButOH. Then, 100 mg of ZIF-8 nanoparticles was suspended in the precursor solution and stirred at RT for 24 h. The resulting product was collected by centrifugation at 9000 rpm for 10 min and washed several times with fresh 1-ButOH under the same conditions. The final crystals were dried and activated at 40 °C overnight. ZIF-8 nanoparticles were synthesized according to a previously reported method^40^ with some modifications. Typically, 1.467 g of Zn(NO_3_)_2_·6H_2_O (4.93 mmol) and 3.245 g of 2-mIm (39.52 mmol) were dissolved in 150 mL of MeOH. Once dissolved, the ligand solution (2-mIm) was poured into the metal solution under stirring. The resulting solution was further stirred for 30 min at RT followed by centrifugation at 9000 rpm for 10 min and washing with fresh MeOH. The final product was dried and activated at 40 °C overnight. This SALE method allowed the synthesis of ZIF-94 with the narrow particle size distribution of ZIF-8 (ca. 45 nm) and the high CO_2_-philicity of ZIF-94.

#### 2.2.3 Preparation of TFC and TFN Membranes

First, polysulfone (PSF) supports were prepared by phase inversion.^41^ Briefly, a 15 wt % doped solution was prepared by dissolving PSF pellets in NMP under stirring overnight at RT. Once dissolved, the solution was degassed for 1 h. After that, the polymer solution was cast on a Teflon plate using an Elcometer 4340 automatic film applicator at a thickness of 250 μm and a casting speed of 0.05 m·s^–1^. Membranes prepared this way were immersed in a water bath for 1 h at RT for polymer precipitation and then transferred to a deionized water bath, where they remained overnight. Finally, the membranes were rinsed with 2-propanol and dried at 40 °C for 24 h.

To avoid penetration of the selective layer into the support porosity, a gutter layer of PTMSP was spin-coated (Laurell Technologies Corp., model WS-650MZ-23NPP/A1/AR1) onto the PSF support. A PTMSP solution was prepared by dissolving the polymer in *n*-hexane in a concentration of 2 wt %. After that, 0.7 mL of the PTMSP solution was spin-coated on top of the PSF support at 2500 rpm during 20 s. Supports with the gutter layer were introduced into an oven at 40 °C for 2 h for complete solvent evaporation.

Finally, the selective layer of Pebax 1657 was spin-coated onto the PTMSP/PSF supports under the same conditions used for the PTMSP layer. For this purpose, a Pebax 1657 solution was prepared dissolving under reflux 0.25 g of the Pebax in 4.75 g of an EtOH/H_2_O (70/30 v/v) mixture at 90 °C for 2 h. Once dissolved and cooled down to RT, 0.6 mL of the Pebax solution was poured onto the PTMSP/PSF support and spun to obtain the Pebax 1657/PTMSP/PSF TFC membrane. In the case of Pebax/UiO-66/ZIF-94 TFN membranes, the polymer was dissolved in 2/3 of the total solvent under the same conditions, while different amounts (accounting for 5–10 wt % of the filler, with respect to the polymer) of UiO-66, UiO-66-NO_2_, and UiO-66-NH_2_ suspensions and 5–15 wt % (with respect to the polymer) of ZIF-94 (if used it) (see Table 1) were mixed with the remaining solvent (1/3 of the total) using an ultrasonic bath (Ultrasons H-D, Selecta). It is worth mentioning here that in order to achieve an efficient gas separation performance, ZIF-94 must be added in higher weight percentages than UiO-66 nanoparticles. Once the polymer was dissolved, the UiO-66 suspensions were added to the Pebax solution and stirred for 1 h before spinning. After spinning, all the membranes were placed in an oven at 40 °C for 18 h to remove any residual solvent.

**Table 1:** MOF Percentages of the Prepared Membranes

**membrane**	**UiO-66**	**wt %UiO-66**	**wt %ZIF-94**
TFC_P1657		0	0
TFN_U(5)	UiO-66	5	0
TFN_U(7.5)	UiO-66	7.5	0
TFN_U(10)	UiO-66	10	0
TFN_UNH2(5)	UiO-66-NH_2_	5	0
TFN_UNH2(7.5)	UiO-66-NH_2_	7.5	0
TFN_UNH2(10)	UiO-66-NH_2_	10	0
TFN_UNO2(5)	UiO-66-NO_2_	5	0
TFN_UNO2(5)_Z94(5)	UiO-66-NO_2_	5	5
TFN_UNO2(5)_Z94(10)	UiO-66-NO_2_	5	10
TFN_UNO2(5)_Z94(15)	UiO-66-NO_2_	5	15

#### 2.2.4 MOF and Membrane Characterization

Scanning electron microscopy (SEM) images of ZIF-94 and membranes were obtained by using a field-emission SEM (FE-SEM, Inspect F50, Thermo Fisher Scientific) operated at 10 kV. This instrument was also used for measuring the thickness of the gutter and selective layers. Cross sections of membranes were prepared by freeze-fracturing after immersion in liquid N_2_. Samples were then mounted on a stub with carbon tape and subsequently coated with Pd (14 nm). TEM imaging of the cross section of the membrane TFN_UNO2(5)_Z94(10) (i.e., 5 wt % UiO-66-NO_2_ and 10 wt % ZIF-94) was performed using a Tecnai T20 (Thermo Fisher Scientific, formerly FEI) operated at an accelerating voltage of 200 kV in order to find out in detail each layer thickness, structure, and arrangement. For this purpose, the membrane was embedded in epoxy resin EMbed 812 at 60 °C for 48 h. After epoxy polymerization, the sample was ultrathin sectioned, using an ultramicrotome Leica EM UC7, to slices of 70 nm in thickness, and they were directly deposited over a carbon film on a 200 mesh copper grid. Chemical information from these sections was acquired by means of X-ray spectrometry (EDS) using a probe aberration-corrected Titan low-base transmission electron microscope (Thermo Fisher Scientific) at a working voltage of 300 kV in the scanning transmission electron microscopy (STEM) mode. The microscope was fitted with a silicon drift detector (SDD) Oxford energy-dispersive X-ray spectrometer. Thermogravimetric analyses (TGA) were carried out using a Mettler Toledo TGA/STDA 851e. Small pieces of membranes (∼3 mg) placed in 70 μL alumina pans were heated under an airflow (40 cm^3^ (STP) min^–1^) from 35 to 700 °C at a heating rate of 10 °C min^–1^. ZIF-94 and membrane crystallinity were analyzed by powder X-ray diffraction (PXRD) using PANalytical Empyrean equipment with Cu Kα radiation (λ = 1.5418 Å) over the range of 5–40° at a scan rate of 0.03° s^–1^. PXRD on a transmission mode-based high-throughput Bruker D8 Advance diffractometer (λ = 1.5418 Å) was also used for structure confirmation of UiO-66-based MOFs. Both TGA and XRD experiments were carried out with dense membranes prepared by casting solution with the remaining Pebax/MOF coating solution. High-resolution TEM images (HRTEM) of MOF nanoparticles were acquired on a Titan Themis 200 microscope operating at 200 kV. This microscope was equipped with a Ceta 16 M hybrid camera from Thermo Fisher Scientific capable of working under low electron irradiation conditions. The N_2_ adsorption isotherms of the MOFs prepared were measured using a Micromeritics Tristar 3000 at −196 °C. Prior to the isotherm measurement, the samples were degassed for 8 h under vacuum at 200 °C, using a heating rate of 10 °C min^–1^. The specific surface area (SSA) of the porous materials was calculated using the Brunauer–Emmett–Teller (BET) method.

#### 2.2.5 Gas Separation Tests

Membranes were cut and placed in a module consisting of two stainless-steel pieces and a 316LSS macroporous disk support (Mott Co.) with a 20 μm nominal pore size. Membranes, 2.12 cm^2^ in area, were gripped inside with Viton O-rings. To control the temperature of the experiment (35 °C), the permeation module was placed in a UNE 200 Memmert oven. The gas separation measurements were carried out by feeding the postcombustion gaseous mixture CO_2_/N_2_ (15/85 cm^3^ (STP) min^–1^) and the mixture CO_2_/CH_4_ (50/50 cm^3^ (STP) min^–1^) to the feed side at an operating pressure of 3 bar to favor CO_2_ permeation.^41^ Gas flows of the mixtures were controlled by mass flow controllers (Alicat Scientific, MC-100CCM-D). The permeate side of the membrane was swept with a 50 cm^3^ (STP) min^–1^ of He, at atmospheric pressure (∼1 bar) (Alicat Scientific, MC-100CCM-D). Concentrations of CO_2_, N_2_, and CH_4_ in the outgoing streams (permeate side) were analyzed online by an Agilent 990 MicroGC. Permeances of CO_2_, N_2_, and CH_4_ were calculated in a GPU (gas permeance unit, 10^–6^ cm^3^ (STP) cm^–2^ s^–1^ cmHg^–1^), once the steady state of the exit stream was reached. The CO_2_/N_2_ and CO_2_/CH_4_ separation selectivities were calculated as the ratios of the corresponding permeances. At least three different membrane samples prepared in the same conditions were tested to calculate the error bars shown.

## 3 Results

### 3.1 MOF Characterization

**Figure 1:**
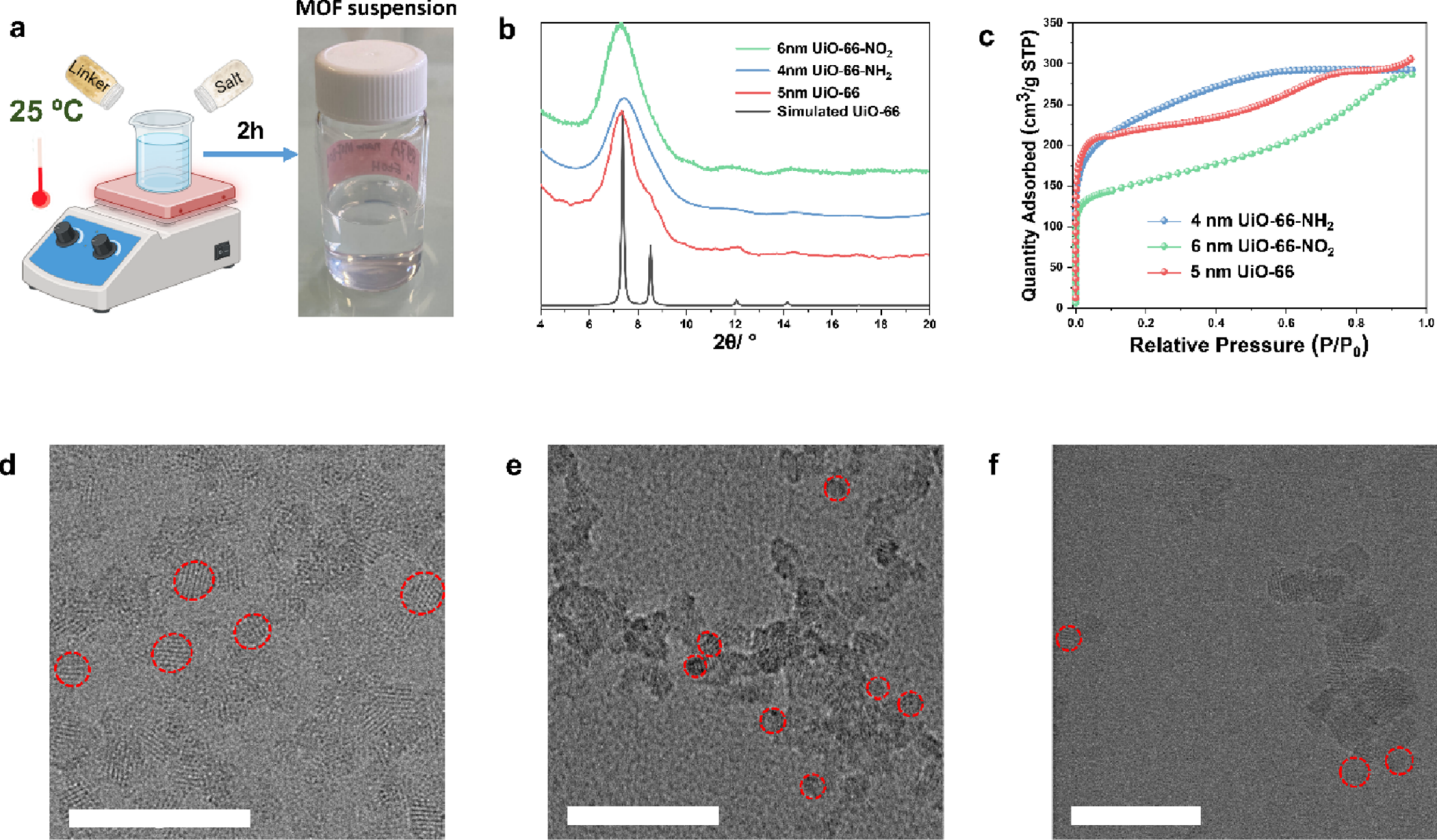
Scheme of the typical synthesis method in this report; the photograph shows the synthetic solution of ultrasmall UiO-66 (a); PXRD patterns of the synthesized ultrasmall UiO-66, UiO-66-NH_2_, and UiO-66-NO_2_ and the corresponding simulated PXRD pattern (b); N_2_ sorption isotherms at −196 °C of the ultrasmall UiO-66, UiO-66-NH_2_, and UiO-66-NO_2_ (c); HRTEM images of 5 nm UiO-66 (d), 4 nm UiO-66-NH_2_ (e), and 6 nm UiO-66-NO_2_ (f); scale bar = 50 nm. For clarity, the red circumferences in parts d–f surround some selected ultrasmall MOF nanoparticles.

The UiO-66, UiO-66-NH_2_, and UiO-66-NO_2_ nanoparticles were prepared through a new stepwise room-temperature strategy developed by some of us^32^ (Figure 1a), where the presynthesized Zr_6_O_4_(OH)_4_ oxoclusters were required. The prerequisite Zr_6_ oxoclusters were prepared successfully according to the identical powder X-ray diffraction (PXRD) pattern (Figure S1) compared to the literature.^42^ The PXRD patterns of the synthesized UiO-66, UiO-66-NH_2_, and UiO-66-NO_2_ show very broad Bragg peaks (Figure 1b), suggesting the absence of long-range order. These broad peaks observed basically coincide with those simulated for the structure of UiO-66. This is often related to the formation of either the amorphous phase or the ultrasmall crystals. The N_2_ isotherms in Figure 1c reveal the high porosity of the synthesized materials, with BET specific surface areas of 875 (±4), 842 (±4), and 586 (±3) m^2^·g^–1^ for UiO-66, UiO-66-NH_2_, and UiO-66-NO_2_, respectively. These values indicate the high quality of the ultrasmall MOFs despite the amorphous-like PXRD patterns. Subsequently, high-resolution transmission electron microscopy (HRTEM) was performed to confirm the size and crystallization of MOFs. As shown in Figure 1d–f, the HRTEM images evidence the formation of very well-crystallized (crystal lattices) and ultrasmall MOF nanoparticles. The prepared UiO-66, UiO-66-NH_2_, and UiO-66-NO_2_ were single crystals with average sizes between 4 and 6 nm (Figure S2, particle size statistical histogram), in line with the broad PXRD peaks mentioned above.

ZIF-94 particles were synthesized from ZIF-8 crystals via a SALE reaction. As observed in Figure S3a, the average particle size of synthesized ZIF-94 is 45 nm. This image further emphasizes the homogeneity of the size distribution. By using this method, ZIF-94 is produced with the narrow particle size distribution of ZIF-8 and the strong CO_2_-philicity of ZIF-94 while avoiding the use of other harmful solvents like tetrahydrofuran (THF) or dimethylformamide (DMF).^43^ The crystallinity and purity of the ZIF particles were confirmed by PXRD. The patterns of the simulated ZIF and synthesized ZIF-94 are plotted together for comparison in Figure S3b. As seen in this figure, the peak positions match well with those of the simulated ZIF-94 (SOD type structure).

### 3.2 Membrane Characterization

**Figure 2:**
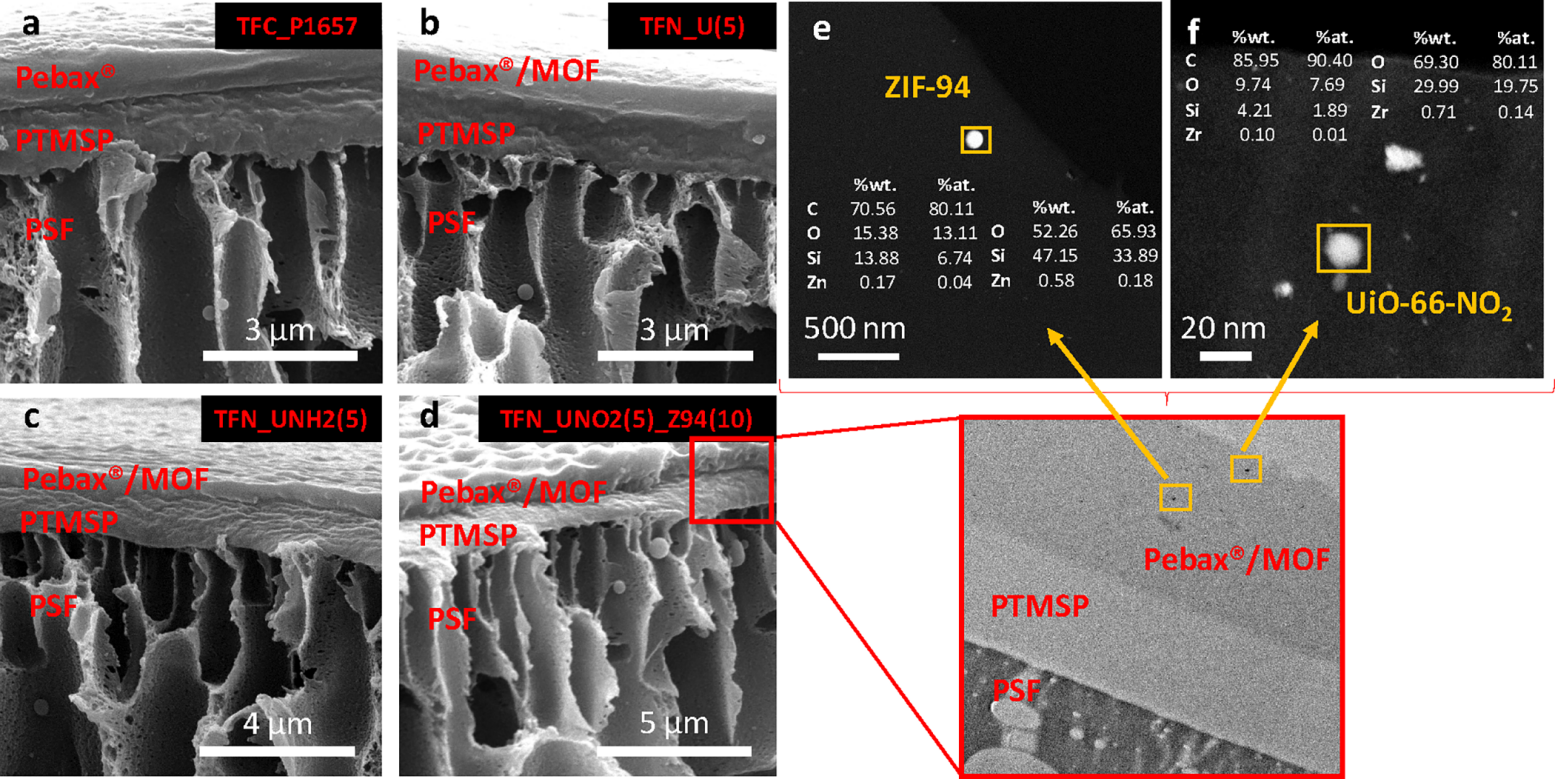
SEM cross-sectional images of the Pebax TFC membrane (a) and the TFN membranes prepared with 5 wt % UiO-66 (b), 5 wt % UiO-66-NH_2_ (c), and 5 wt % UiO-66-NO_2_ and 10 wt % ZIF-94 (d). The inset corresponds to a TEM cross-sectional image of the Pebax TFN membranes prepared with 5 wt % UiO-66-NO_2_ and 10 wt % ZIF-94. High-angle annular dark-field–scanning transmission electron microscopy (HAADF–STEM) and energy-dispersive X-ray spectroscopy (EDX) of the corresponding Zn (e) and Zr (f) scans.

Cross sections of the TFC (only polymer) and TFN (including nanoparticles in the skin layer) membranes were explored by SEM and are depicted in Figure 2a–d. Membranes show three different layers, corresponding to the PSF support, the PTMSP gutter layer, and the selective skin layer (Pebax or Pebax embedding MOF nanoparticles). The absence of defects in the selective skin layers of the membrane makes it evident that the type of transport of gaseous species across membranes is the solution-diffusion mechanism. The thicknesses of both the gutter layer and the selective layer were also estimated by SEM, being approximately 1 μm and 600 nm, respectively. From these images, a TEM image of a cross section of the TFN membrane prepared with 5 wt % UiO-66-NO_2_ and 10 wt % ZIF-94 prepared by ultramicrotomy is shown in the zoom of Figure 2d, and it allows to distinguish the two polymer layers on top of the PSF support. This detailed characterization was only carried out on the best performing membranes (shown below), and the image further highlights that the MOF nanoparticles are located exclusively in the Pebax layer and that they are evenly distributed through it. The presence of MOF nanoparticles in the membrane was also confirmed by high-angle annular dark-field–scanning transmission electron microscopy (HAADF–STEM) and energy-dispersive X-ray spectroscopy (EDX). The corresponding Zn and Zr scans are depicted in Figure 2e,f. As expected, Zn and Zr contributions from ZIF-94 and UiO-66-NO_2_, respectively, were detected although their weight composition is below 1 wt %. This is mainly explained by the fact that the percentage of metal in the MOF is low compared to that of the rest of the elements present in the membrane and in the sample prepared for observation (Cl in the resin and C in the MOF, polymers, and in the TEM grid itself). This is the reason that the element percentages are shown with or without the carbon signal to emphasize the metal signal. It must be noted that the Si signal observed in Figure 2e,f comes from the detector itself. In any event, the HAADF–STEM characterization shows that the ultrasmall functionalized UiO-66 is individually present, nonagglomerated in the polymer, just like ZIF-94 nanoparticles.

**Figure 3:**
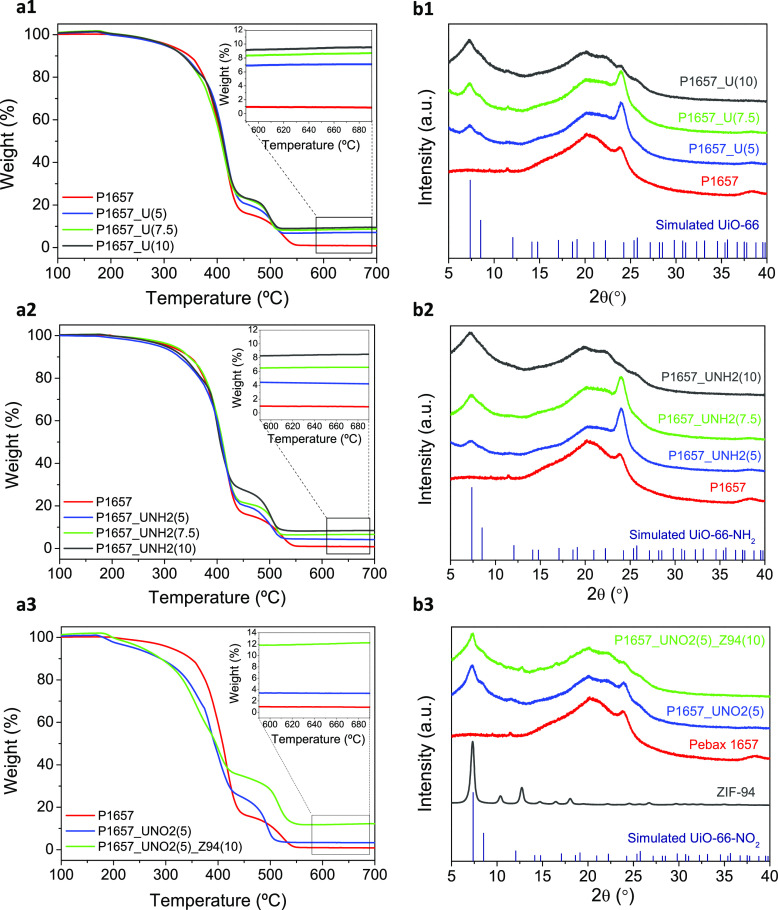
TGA curves (a1–a3, with insets showing the remaining weights corresponding to ZrO_2_ and ZnO) and XRD patterns (b1–b3) of the dense membranes prepared with the remaining casting solution.

The thermal stability and crystallinity of the membrane samples were studied by TGA and PXRD analyses. Due to the huge dilution effect that the membrane support produces, the TFC or TFN membranes were not directly examined by these two techniques but only the materials constituting their thin skin layers. With this aim, dense membranes were prepared by casting the remaining Pebax/MOF solution onto a glass Petri dish and treated under the same conditions (40 °C overnight). Results of TGA and PXRD analyses are depicted in Figure 3a1–a3,b1–b3. As seen in Figure 3a1,a2, the membrane samples fabricated with UiO-66 and UiO-66-NH_2_ are stable up to 350 °C, with no significant weight loss before that temperature, which indicates the successful activation of membranes (i.e., no loss of solvents is appreciated). From this point onward, membranes undergo a sharp degradation until 450 °C. Next, membranes experience a second degradation step, related to the combustion of aromatic compounds,^8^ which continues until 560 °C. At this temperature, the residue of the bare membrane is below 1% of its initial weight. This residue increases with the concentration of the MOF in the polymer matrix due to the ZrO_2_ and ZnO generated during the thermal oxidation step.^44,45^ In the particular case of the membranes fabricated with UiO-66-NO_2_ (Figure 3a3), their thermal degradation starts at a lower temperature (220 °C), which suggests that the thermal stability of these samples is highly affected by the incorporation of the −NO_2_-functionalized UiO-66 particles, which show significant weight losses at 400 °C due to ligand decomposition.^46^ The PXRD patterns depicted in Figure 3b1–b3 reveal that the MOFs did not lose their structure upon incorporation in the membranes and the increase of peak intensity with the amount of the MOF, which is evidenced by the intensification of the Bragg peak at a 2θ value of 7.3°, related to both UiO-66 (with and without functionalization) and ZIF-94.^47^ In contrast, the peak at 2θ = 24.4° corresponding to the Pebax polymer^48^ decreases at a high loading (10 wt %), suggesting that the MOF particles hinder the entanglement of the polymer chains.

### 3.3 Gas Separation Tests

**Figure 4:**
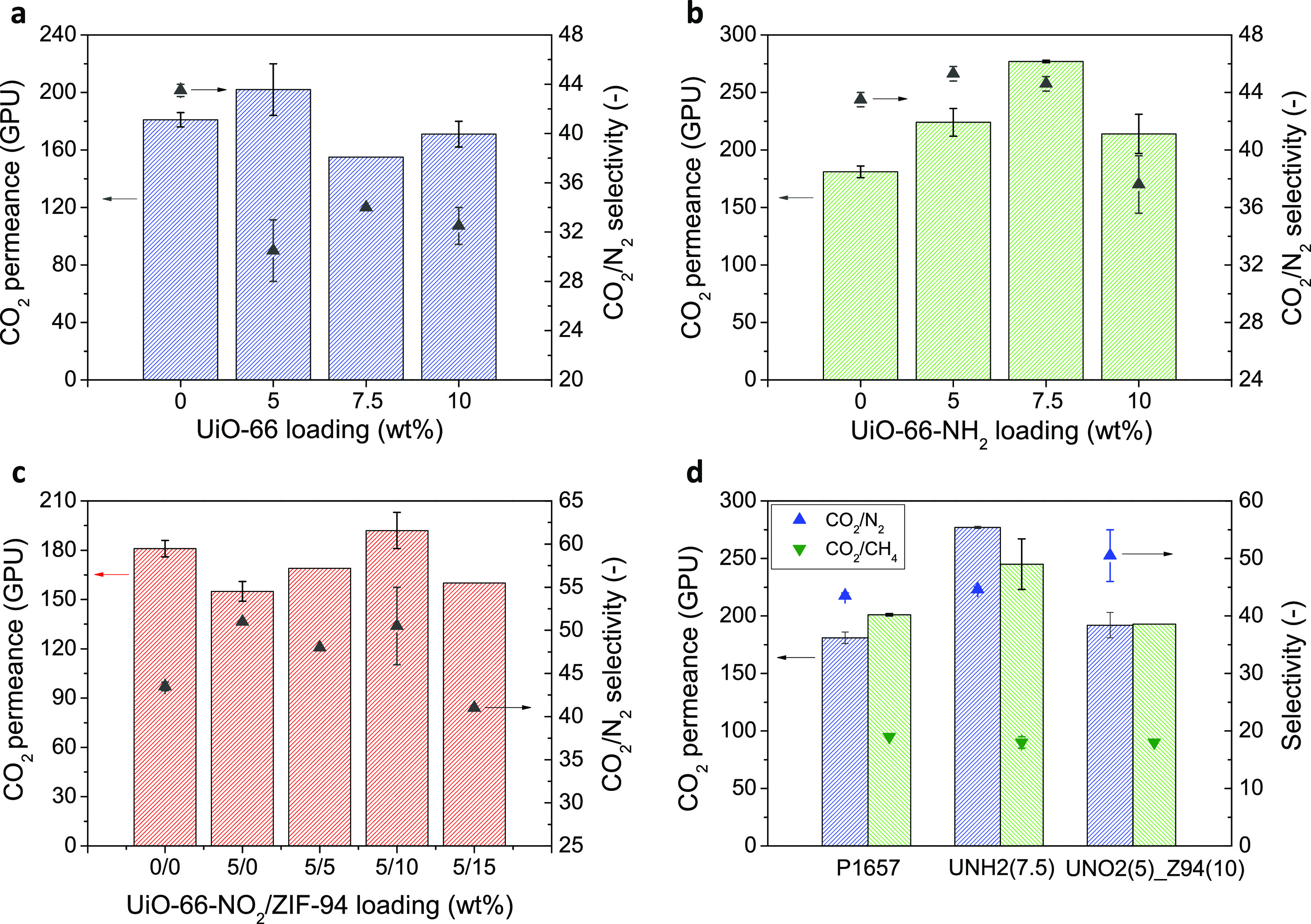
CO_2_/N_2_ separation performance at 35 °C and 3 bar of the TFC and TFN membranes fabricated with UiO-66 (a), UiO-66-NH_2_ (b), and UiO-66-NO_2_ and ZIF-94 (c) and a comparison of both CO_2_/N_2_ and CO_2_/CH_4_ separation performance of the membranes prepared with the best conditions (d).

As shown below, due to the potential agglomeration of the ultrasmall UiO-66 MOFs, nanoparticles of a second filler were simultaneously added to the same TFN membrane. This is ZIF-94, chosen because of its CO_2_-philicity^49^ and also its different composition and structure able to establish a synergy with UiO-66. Therefore, to achieve an efficient membrane separation performance, ZIF-94 must be added in weight percentages higher than those corresponding to UiO-66. Interestingly, it has been shown previously that the combination of two MOFs with different characteristics (in this case, one more hydrophilic, ZIF-94, and the other more hydrophobic, UiO-66) in a membrane can lead to a synergistic effect on the separation of mixtures with CO_2_ due to the fact that the filler dispersion is improved by avoiding its agglomeration.^35^ Having said this, the CO_2_/N_2_ gas separation was studied, at 35 °C and 3 bar feed pressure, first with the TFN membranes fabricated with UiO-66 (Figure 4a) and UiO-66-NH_2_ (Figure 4b) and then with UiO-66-NO_2_ + ZIF-94 (Figure 4c). For comparison purposes, the bare Pebax 1657 TFC membrane was also studied. Moreover, the best conditions found for the CO_2_/N_2_ separation have been compared and tested for the CO_2_/CH_4_ tests (Figure 4d). The results plotted in Figure 4 are collected as well in Tables S1–S4 of the SI. As seen in Figure 4a and Table S1, the addition of 5 wt % of the bare UiO-66 nanoparticles increases the CO_2_ permeance from 181 to 202 GPU, although the CO_2_/N_2_ selectivity decreases from 43.5 to 30.5. At higher loadings, the CO_2_ permeance is below the value obtained with the bare TFC membrane. These results suggest a limitation in the compatibility between this MOF and the Pebax 1657 polymer. As expected, the introduction of functional groups within the UiO-66 structure enhances the MOF–polymer compatibility due to the hydrogen bonds created between the polymer chains (with N and O electronegative atoms bonded to hydrogens) and the functional group.^50^ As observed in Figure 4b,c and Tables S2 and S3, this allows obtaining the highest CO_2_ permeance with 7.5 wt % UiO-66-NH_2_ (277 GPU), without affecting the CO_2_/N_2_ separation selectivity (44.6). Furthermore, the increase in the CO_2_-philicity due to the introduction of amino groups in the MOF structure may also be responsible of such an enhancement.^14^ Nevertheless, increasing the amount of UiO-66-NH_2_ in the polymer matrix above 7.5 wt % is translated into a decrease of both the CO_2_ permeance and CO_2_/N_2_ selectivity, probably due to the agglomeration of the nanoparticles inside the membrane.^51^ Similarly, with the UiO-66-NO_2_ nanoparticles, the optimal loading is 5 wt %. Unlike the bare UiO-66 and UiO-66-NH_2_, which were dispersed in the mixture EtOH/water, the nanoparticles of this MOF were initially suspended only in water, making it difficult to dissolve the polymer in the rest of the solvent. Considering the amount of water added with the MOF suspension, the ratio between EtOH and water was recalculated in order to dissolve the Pebax and obtain the final MOF–Pebax solution in the mixture EtOH/water 70/30. With 5 wt % UiO-66-NO_2_, the CO_2_ permeance decreases from 181 to 155 GPU. However, the CO_2_/N_2_ selectivity increases by 17%, from 43.5 to 51.0, being the highest separation selectivity obtained in this work.

In view of this result, 45 nm ZIF-94 particles were added to the UiO-66-NO_2_/Pebax solution with the aim of further increasing the CO_2_ permeance (due to the increase of the total MOF loading in the TFN membrane) while maintaining or increasing the CO_2_/N_2_ selectivity.^48^ As observed in Figure 4c, the CO_2_ permeance of the membranes with both UiO-66-NO_2_ and ZIF-94 reaches a maximum of 192 GPU at 10 wt % ZIF-94 with a maintained CO_2_/N_2_ separation selectivity of ca. 51, the best values in this work. This is related to the properties of MOFs and the synergistic effect that would improve their dispersion, as seen by electronic microscopy. As for the UiO-66-NH_2_ TFN membranes, from this loading, both the CO_2_ permeance and CO_2_/N_2_ selectivity decrease due to particle agglomeration, even though the combination of the two different fillers allowed the highest total effective MOF loading of 15 wt %.

At this point, the best separation conditions were found with the TFN_UNH_2_(7.5) (highest CO_2_ permeance) and TFN_UNO_2_(5)_Z94(10) (highest CO_2_/N_2_ selectivity) membranes. Figure 4d depicts a comparison of the results obtained with these membranes, in which the CO_2_/CH_4_ separation performance is also included. It is worth noting that the gas content in raw natural gas varies accordingly to its geo-origin, and, in addition to CO_2_ and CH_4_, it is composed of a variety of other compounds such as H_2_S, NH_3_, or siloxanes, among others. The presence of those trace pollutants in the natural gas stream can adversely affect the gas separation performance and the long-term stability of the membrane. For example, the presence of H_2_S usually reduces the CO_2_ permeance due to the preferential adsorption of H_2_S in the metal sites of MOFs in MMMs.^37^ Having said this, the aim of this work was to study the effects that ultrasmall nanoparticles have in the separation of CO_2_/CH_4_ gas mixtures, so no content of other gases has been considered so far. However, we believe that additional work should be done to investigate the influence of the presence of trace pollutants on the separation performance of these membranes.

Although the CO_2_/N_2_ selectivity of the TFN_UNO_2_(5)_Z94(10) membrane is higher than those of the TFC_P1657 and TFN_UNH_2_(7.5) membranes, the CO_2_/CH_4_ selectivity does not experience such an enhancement, neither for the CO_2_ permeance nor for the selectivity. In this sense, the only membrane that clearly improves the CO_2_ permeance in CO_2_/CH_4_ separations is the one fabricated with 7.5 wt % UiO-66-NH_2_. This could be due to both the intrinsic gas sorption properties of UiO-66-NH_2_, which have been proven to be above those of UiO-66 and UiO-66-NO_2_ (CO_2_ loading at 0.15 bar and 298 K being 2.37, 2.57, and 4.91 wt % for UiO-66, UiO-66-NO_2_, and UiO-66-NH_2_, respectively^52^), and a better colloidal dispersion of the UiO-66-NH_2_ into the hydrophilic Pebax 1657 matrix due to the higher trend of this MOF to constitute hydrogen bonds (as compared to both UiO-66 and UiO-66-NO_2_).

It is worth mentioning that the ultrasmall MOF nanoparticles, in addition to being the suitable material to prepare very thin selective membranes, allow an optimum effect on the gas separation properties at a lower filler concentration than larger UiO-66-based fillers (typically working in the 10–50 wt % range composing in turn thicker membranes).^53,54^ This would allow a reduction of the membrane cost in an eventual large-scale production. Therefore, to confirm the effect of the particle size on the gas separation performance, TFN membranes were also fabricated with 7.5 wt % UiO-66-NH_2_ with an average particle size of 150 nm. Figure S4 shows that the improvement of the gas separation performance of the membranes prepared with larger UiO-66-NH_2_ particles is not as significant as that obtained with the smaller particles, suggesting that higher loadings of large UiO-66-NH_2_ particles are required to achieve comparable gas separation properties. This can be due to the fact that large particles have a lower external surface area, which is translated into a weakened interaction between the filler and the polymer matrix.^55^ Small particles tend to agglomerate, but the design of their composition and morphology is crucial to prevent it,^56^ together with their proper formulation in the TFN membrane. This seems to have been accomplished with the functionalized UiO-66 ultrasmall nanoparticles prepared here. In consequence, as shown in Table S5, the CO_2_ permeance of the TFN_UNH_2_(7.5)_large membrane (205 GPU) is 12% higher than that of the bare TFC_P1657 membrane (181 GPU). Moreover, the CO_2_/N_2_ selectivity value decreases from 43.5 to 38.0 when large UiO-66-NH_2_ particles are used. However, when using the ultrasmall UiO-66-NN_2_, the CO_2_ permeance increases to 277 GPU (a 53% increase) achieving a CO_2_/N_2_ selectivity of 44.6. As reported elsewhere,^57,58^ a high specific surface area contributes to increasing the CO_2_/N_2_ selectivity due to the increase in the CO_2_ capture active sites and the larger N_2_ mass transfer resistance. Additionally, it is worth mentioning that ultrasmall particles allow achieving both high CO_2_ permeance and CO_2_/N_2_ selectivity with the advantage of requiring a lower MOF mass density (0.036 g m^–2^) than larger particles since higher loadings of large UiO-66-NH_2_ particles should be added to the membrane in order to obtain comparable gas separation results, as mentioned before.

A comparison of the gas separation performance of the membranes prepared in this work with that of other Pebax 1657-based TFN membranes found in the literature is collected in Table 2. As seen in this table, the CO_2_/N_2_ separation selectivity achieved in this work falls within the range of values found in the literature, the CO_2_ permeance in the case of the membrane fabricated with UiO-66-NH_2_ (277 GPU) being higher than those of other membranes found in the literature and fabricated with Cu-BTC fillers,^59^ which have similar selectivity values (47.6 and 53.8 for Cu-BTC and Cu-BTC-NH_2_, respectively, vs 44.6, 51.0, and 50.5 for UiO-66-NH_2_, UiO-66-NO_2_, and UiO-66-NO_2_/ZIF-94, respectively). In the case of CO_2_/CH_4_ selectivity, despite the high CO_2_ permeance achieved with the membrane TFN_UNH_2_(7.5) (245 GPU), another work has reported a better separation performance with the same filler (37.5 of CO_2_/CH_4_ selectivity at a CO_2_ permeance of 373 GPU).^29^

**Table 2:** Comparison with Other Pebax 1657 TFN Membranes Containing Different MOF Fillers

MOF	temperature (°C)	pressure (bar)	CO_2_ permeance (GPU)	CO_2_/N_2_ selectivity	CO_2_/CH_4_ selectivity	ref.
UiO-66	35	5	11.5		55.5	(48)
MOF-801	20	1	22.4	66.0		(45)
MIL-101(Cr)-TEPA	25	4	19.4		46.3	(60)
UiO-66	25	5	340.0		30.3	(29)
UiO-66-NH_2_	25	5	373.0		37.5	(29)
ZIF-8	25	2	350.0	31.0	13.0	(61)
ZIF-7	20	4	111.0	30.0	97.0	(62)
Cu-BTC	30	6	228.6	47.6	31.8	(59)
Cu-BTC-NH_2_	30	6	258.3	53.8	38.0	(59)
UiO-66-NH_2_	25	6	328.0		27.0	(63)
UiO-66-NH_2_	35	3	277.0	44.6		this work
UiO-66-NO_2_	35	3	155.0	51.0		this work
UiO-66-NO_2_/ZIF-94	35	3	192.0	50.5		this work
UiO-66-NH_2_	35	3	245.0		18.0	this work
UiO-66-NO2/ZIF-94	35	3	193.0		18.0	this work

## 4 Conclusions

Pebax 1657 TFN membranes based on ultrasmall 4–6 nm nanoparticles of UiO-66 have been successfully fabricated by spin-coating with robust and selective skin layer thicknesses of around 700 nm. The functionalization of MOF UiO-66 with amino (−NH_2_) and nitro (−NO_2_) groups significantly enhances the gas separation performance of the membranes due to the enhancement of the CO_2_ interaction and the increase in the MOF/polymer compatibility derived from the hydrogen bonds created between the polymer chains and the MOF functional groups. The highest CO_2_ permeance was obtained with the TFN membrane containing 7.5 wt % UiO-66-NH_2_, which improved by 47% the CO_2_ permeance of the TFC membrane in the case of CO_2_/N_2_ separations and by 22% in CO_2_/CH_4_ mixtures. Above a 7.5 wt % loading, UiO-66-NH_2_ nanoparticles create agglomerates that hinder the diffusion of CO_2_ through the membrane, which, in turn, decreases the CO_2_ permeance. Furthermore, such agglomerates generate microdefects that entail a reduction of separation selectivity. TFN membranes containing 5 wt % UiO-66-NO_2_ reached the highest value of CO_2_/N_2_ selectivity (51), although the CO_2_ permeance decreases by 14% in comparison to the TFC membrane. To maintain the CO_2_/N_2_ selectivity of 51 and increase the CO_2_ permeance, UiO-66-NO_2_ and ZIF-94 nanoparticles of 4–6 and 45 nm, respectively, were combined in the same TFN membrane to achieve a synergistic effect that improves filler dispersion. TEM observation proved that both UiO-66 and ZIF-94 nanoparticles are located in the selective top layer of the TFN membrane and that they are uniformly distributed through it without agglomeration. This allowed achieving a CO_2_ permeance of 192 GPU, close to that obtained with the pristine TFC membrane (181 GPU) but with a higher CO_2_/N_2_ selectivity (51.0 vs 43.5). Finally, to corroborate the effect of particle size in the gas separation performance, TFN membranes were fabricated with larger UiO-66-NH_2_ particles of 150 nm. As expected, the improvement in the gas separation performance was not as significant as that obtained with the ultrasmall nanocrystals due to the reduction of the MOF specific surface area. Finally, even if some other important aspects (e.g., the presence of minor components in the feed, temperature and pressure conditions, and long-term operation, the latter already studied with good prospect with analogous Pebax Rnew TFN membranes in our previous publication^36^) could have been addressed as well, this work paves the way to a new generation of TFN membranes based on the use of lower amounts of ultrasmall MOF nanoparticles able to produce an increase in the gas separation performance at a relatively low filler loading, and furthermore, the combination of different types of MOFs in a TFN membrane can produce synergistic effects increasing the gas separation performance.

## Supporting Information Available

XRD of the Zr_6_ oxocluster; particle size distributions of ultrasmall UiO-66 MOFs; SEM and XRD of ZIF-94; tables containing CO_2_/N_2_ and CO_2_/CH_4_ gas separation performance values of TFN membranes; CO_2_/N_2_ separation performance of TFN membranes prepared with UiO-66-NH_2_ of different sizes (PDF)
