# Supplementary material for: Ultrasmall Functionalized UiO-66 Nanoparticle/Polymer Pebax 1657 Thin-Film Nanocomposite Membranes for Optimal CO2 Separation
Source: ACS Appl Mater Interfaces. 2024 Jan 12;16(3):4024–34. doi: 10.1021/acsami.3c16093 (PMC10811625; doi:10.1021/acsami.3c16093)
Supplement: Supplementary file 1 — am3c16093_si_001.pdf [file am3c16093_si_001.pdf]

Ultra-small functionalized UiO-66 nanoparticles/polymer Pebax® 1657 thin film nanocomposite membranes for optimal CO_2 separation

Lidia Martínez-Izquierdo[1,2], Cristina García-Comas[1,2], Shan Dai[4], Marta Navarro[1,3], Antoine Tissot[4], Christian Serre[4], Carlos Téllez[1,2] and Joaquín Coronas[1,2*]

[1]Instituto de Nanociencia y Materiales de Aragón (INMA), Universidad de Zaragoza-CSIC, 50018 Zaragoza. Spain

[2]Chemical and Environmental Engineering Department, Universidad de Zaragoza, 50018 Zaragoza. Spain

[3]Laboratorio de Microscopías Avanzadas, Universidad de Zaragoza, 50018 Zaragoza.

[4]Institut des Matériaux Poreux de Paris, Ecole Normale Supérieure, ESPCI Paris, CNRS, PSL University, 75005, Paris, France

[*]Corresponding author e-mail address: coronas@unizar.es, christian.serre@ens.fr

UiO-66 based materials characterization

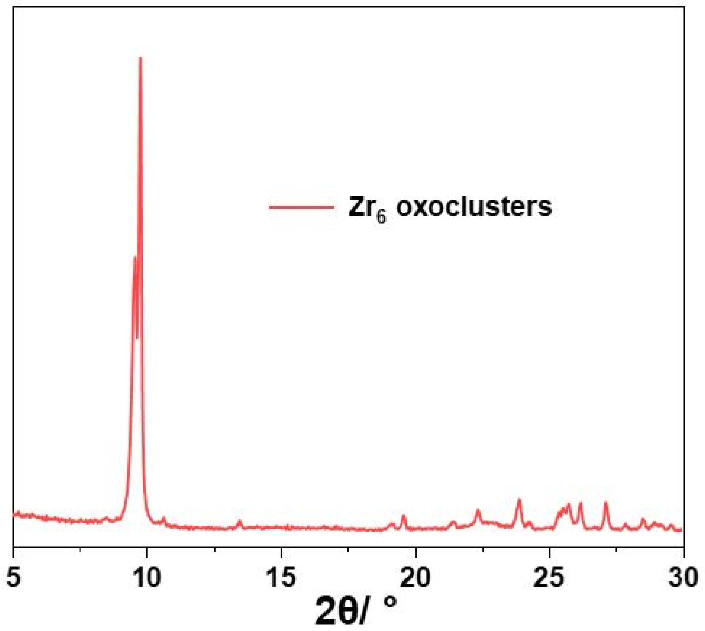

Figure S1. PXRD pattern of the synthesized Zr_6 oxoclusters.

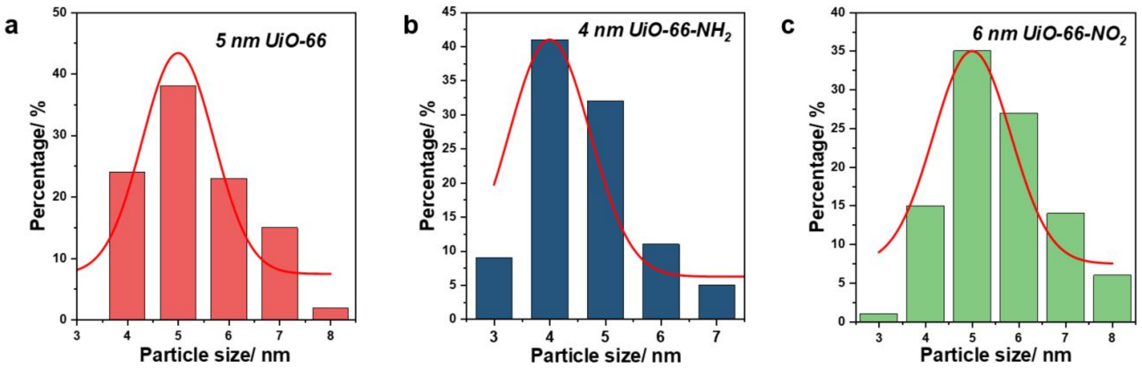

Figure S2. HRTEM particle size distributions of: UiO-66 (a), UiO-66-NH_2 (b), and UiO-66-NO_2 (c).

ZIF-94 characterization

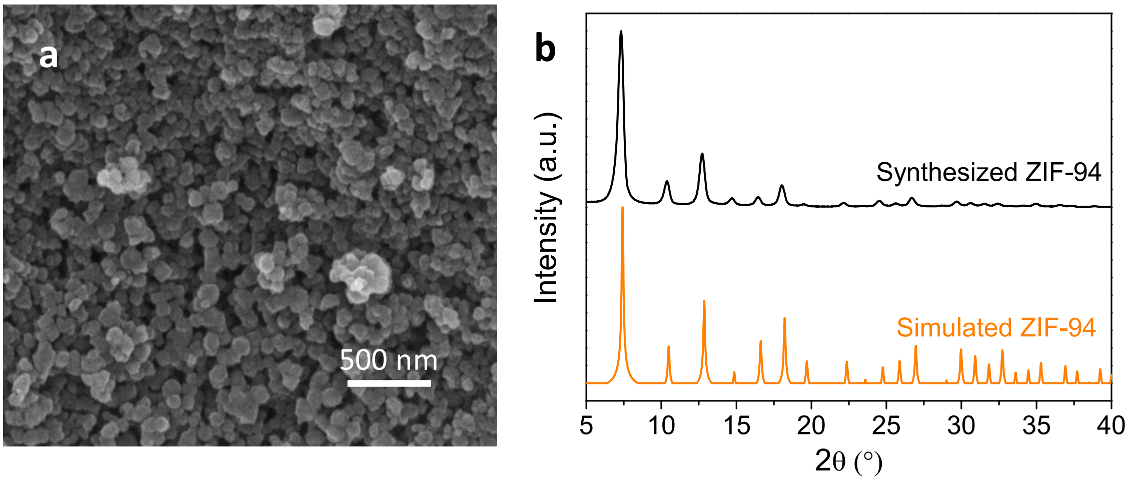

Figure S3. SEM image of ZIF-94 crystals (average particle size = 45 nm) obtained from ZIF-8 by the SALE (solvent assisted ligand exchange) method (a), XRD patterns of the simulated and synthesized ZIF-94 (b).

Gas separation performance

Table S1. CO_2/N_2 separation results of the TFN membranes prepared with Pebax® 1657 and UiO-66 at 35 °C and 3 bar.

Membrane	CO_2 permeance<br>GPU	CO_2/N_2 selectivity<br>(-)
**TFC_P1657**	181 ± 5	43.5 ± 0.5
**TFN_U(5)**	202 ± 18	30.5 ± 2.5
**TFN_U(7.5)**	155 ± 0	34.0 ± 0.0
**TFN_U(10)**	171 ± 9	32.5 ± 1.5

Table S2. CO_2/N_2 separation results of the TFN membranes prepared with Pebax® 1657 and UiO-66-NH_2 at 35 °C and 3 bar.

Membrane	CO_2 permeance<br>GPU	CO_2/N_2 selectivity<br>(-)
**TFC_P1657**	181 ± 5	43.5 ± 0.5
**TFN_UNH2(5)**	224 ± 12	45.3 ± 0.5
**TFN_UNH2(7.5)**	277 ± 1	44.6 ± 0.5
**TFN_UNH2(10)**	214 ± 17	37.6 ± 2.0

Table S3. CO_2/N_2 separation results of the TFN membranes prepared with Pebax® 1657, UiO-66-NO_2 and ZIF-94 at 35 °C and 3 bar

Membrane	CO_2 permeance	CO_2/N_2 selectivity
	GPU	(-)
**TFC_P1657**	181 ± 5	43.5 ± 0.5
**TFN_UNO2(5)**	155 ± 6	51.0 ± 0.0
**TFN_UNO2(5)_Z94(5)**	169 ± 0	48 ± 0.0
**TFN_UNO2(5)_Z94(10)**	192 ± 11	50.5 ± 4.5
**TFN_UNO2(5)_Z94(15)**	160 ± 0	41.0 ± 0.0

Table S4. CO_2/N_2 and CO_2/CH_4 separation results of the best TFN membranes prepared in this work. Measured at 35 °C and 3 bar

Membrane	CO_2/N_2		CO_2/CH_4	
	CO_2 permeance	selectivity	CO_2 permeance	selectivity
	GPU	(-)	GPU	(-)
**TFC_P1657**	181 ± 5	43.5 ± 0.5	201 ± 1	19.0 ± 0.0
**TFN_UNH2(7.5)**	277 ± 1	44.6 ± 0.5	245 ± 22	18.0 ± 1.0
**TFN_UNO2(5)_Z94(10)**	192 ± 11	50.5 ± 4.5	193 ± 0.0	18.0 ± 0.0

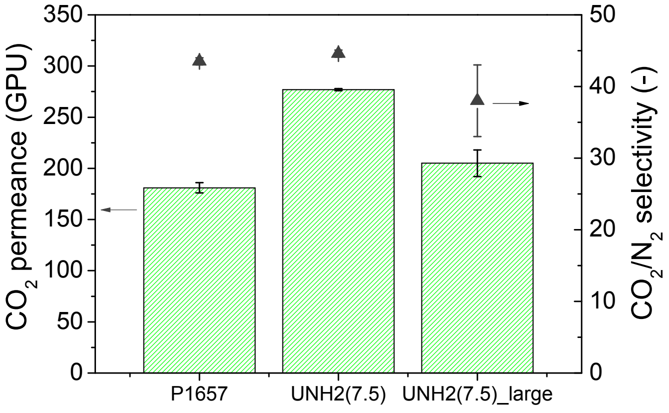

Figure S4. CO_2/N_2 separation performance of the TFC and TFN membranes fabricated with 7.5 wt% of UiO-66-NH_2. NH_2 at 35 °C and 3 bar. Influence of the particle size.

Table S5. Comparison of the CO_2/N_2 separation results obtained with the TFN membranes prepared with Pebax® 1657 and UiO-66-NH_2 at 35 °C and 3 bar. Influence of particle size.

Membrane	CO_2 permeance	CO_2/N_2 selectivity
	GPU	(-)
**TFC_P1657**	181 ± 5	43.5 ± 0.5
**TFN_UNH2(7.5)**	277 ± 1	44.6 ± 0.5
**TFN_UNH2(7.5)_large**	205 ± 13	38.0 ± 5.0
